# An R Shiny App for a Chronic Lower Back Pain Study, Personalized N-of-1 Trial

**DOI:** 10.1162/99608f92.6c21dab7

**Published:** 2022-09-08

**Authors:** Thevaa Chandereng

**Affiliations:** Mailman School of Public Health, Columbia University, New York, NY, USA

**Keywords:** personalized medicine, fitbit data, imputation, computing platforms

## Abstract

The call for personalized medicine highlights the need for personalized (N-of-1) trials to find what treatment works best for individual patients. Conventional (between-subject) randomized controlled trials (RCT) yield effects for the ‘average patient,’ but a personalized trial administers all treatments within-subject, so benefits or harms to the individual patient can be identified. The design and analysis of personalized trials involve different strategies from the conventional RCT. These include how to adjust for any carryover effects from one intervention to another, how to handle missing data, and how to provide patients with insight into their data. In addition, a comprehensible report about trial results should be created for each patient and their clinician to facilitate their decision-making. This article describes strategies to address these design and analytic issues, and introduces an R shiny app to facilitate their solution, to explain the use of each of the design and statistical strategies. To illustrate, we also provide a concrete example of a personalized trial series designed to increase activity (i.e., walking steps) in patients with chronic lower back pain (CLBP).

## 1. Introduction

Clinicians often rely on evidence from conventional between-subject randomized controlled trials (RCTs) for guidance to treat their patients. Such trials randomly allocate research participants to two or more treatment arms and/or a control group. At the conclusion of the trial, the average effects for each treatment group are computed and then compared for a measured outcome, such as symptoms, pain, health quality of life, and so on. Assuming there is minimal variability in response to treatment, the best prediction of treatment benefit for an individual patient can be estimated from the overall trial—the main effect. However, more often heterogeneity of treatment effects (HTEs) (i.e., effect variability across patients) is evident among different patients and subgroups participating in conventional RCTs (Gabler et al., 2011)(Guyatt et al., 1988)(Vijan, 2020). This variability often forces clinicians to make educated guesses about the optimal treatment for specific patients (n.d.-a). (Guyatt et al., 1988) proposed a personalized (N-of-1) trial design as an alternative to the conventional RCT to identify the most beneficial therapy for each patient. In a personalized trial, the individual patient is the sole unit and receives all relevant treatment/control conditions successively (Guyatt et al., 1986). By comparing the effects of one or more treatments within-subject, the comparative benefits (or potential harms) provided by each treatment can be discerned. Furthermore, the patients can receive their trial data in partnership with their clinician, so decisions can be made about which treatment to choose going forward.

Besides the basic differences between traditional and personalized trials described above, such trials diverge in other significant ways. The primary endpoint in a conventional RCT is assessed at the end of the study period. In personalized trials, the primary outcome is measured periodically throughout the trial because the outcomes must be measured in response to each treatment. It follows that traditional RCTs typically collect a few outcome measures from many people, whereas a personalized trial collects many, frequent measurements from a single individual. Another difference is that personalized trials are most appropriate for medical conditions or symptoms that should show rapid change when treatments are introduced or withdrawn. Thus, prevention efforts for relieving patients with chronic, stable, or slowly progressive conditions are the best candidates for personalized trials. (Acute or quickly deteriorating conditions are inappropriate, (n.d.-b); irreversible interventions are ruled out because of the within-subject nature of personalized trials.)

Despite their advantages, personalized trials have not received substantial uptake, largely because clinicians and researchers have lacked an automated, sustainable, user-friendly technology platform to permit the facile conduct of such trials. The situation has changed with the recent advent of health apps, texting, remote sensors, and automated platforms that enable the virtual delivery of instructions, interventions, and data collection. These state-of-the-art remote technologies facilitate convenient recruitment, screening, and participation in personalized trials at the patient’s home or work.

The design, implementation, and analysis of personalized trial data require special strategies that differ from those used in conventional RCT design. First, the researcher must know how to statistically power the personalized trial to detect within-subject differences in the effects of each treatment. In contrast to the conventional RCT, the statistical power of a personalized trial refers to the number of assessments and treatment periods for each patient (Freeman, 1989)(Willan & Pater, 1986). Second, the researcher should determine the HTE associated with the type of treatment and patient. The initial step is to ascertain how large a series of personalized (N-of-1) trials should be conducted. Then, a comparison is made about how each patient’s outcomes differ from effects pooled across all patients in the series. Graphical displays or statistical tests of the data can establish the HTEs (Araujo et al., 2016)(Cheung et al., 2020). Procedures to calculate these values will be described below.

Third, special strategies should be adopted, because treatment arms in a personalized trial are not independent; participants receive all treatments. Also, the outcome variable is collected very frequently or continuously throughout the trial. Thus, the outcome data constitute a time series, which should be analyzed using a correlation structure (Shaffer et al., 2018). An additional complication is that exposure to all treatments may produce carryover effects (i.e., when a treatment block influences the outcomes of a subsequent block). One option to minimize carryover effects is to insert no-treatment breaks between different conditions. However, any residual carryover effects may lead to conservative differences in treatment effects because of type-II error inflation (Alemayehu et al., 2017). A statistical strategy, described below, adjusts for carryover effects (Shaffer et al., 2018) and evaluates the effect of each treatment the patient receives on the outcome variable.

Another consideration is that personalized trials require frequent collection of the outcome variables for weeks or months. Both patient non-adherence and technological failures may result in missing data. Some researchers prefer to conduct statistical analyses of the treatment effects even if there are missing data. However, other researchers prefer to use data imputation strategies. Below we will describe an analytic procedure to impute missing data.

Fifth, a major aim of the personalized trial is the provision of feedback to the patient about their results after the trial. This feedback should be reported in an easy-to-comprehend format that permits the patient and their clinician to identify which treatment provided the greatest benefits and fewest side effects. Below, we will describe an R Shiny app that provides a user-friendly, graphical display of the personalized trial results to the patient and their clinician. This app also computes within-subject effects for changes in the outcome as a function of each intervention and time period, adjusts for carryover effects, and imputes missing values, if the researcher prefers that.

To demonstrate the concrete use of these strategies, we describe a series of personalized (N-of-1) trials evaluating the effects of two treatments–yoga and massage–to decrease sedentary behavior in chronic lower back pain patients (CLBP). More than 25% of the U.S. adult population is affected by lower back pain (Deyo et al., 2006), which is the fifth most common cause of physician visits (Khan et al., 2014). CLBP leads to limitations in daily activities and low levels of physical exercise (Chou, 2010). For example, a common consequence of CLBP is a significant reduction in everyday walking. A substantial body of epidemiological evidence shows that physical inactivity is a risk factor for cardiovascular diseases, stroke, and so on.

To manage their pain, those with CLBP often take medication; however, recent clinical guidelines recommend less use of pharmacotherapy for CLBP management (Qaseem et al., 2017) because of the dangers of addiction and accidental overdose of opioid treatment (Dowell et al., 2016). The Centers for Disease Control and Prevention (CDC) have called for alternative approaches to manage chronic pain. Both yoga and massage have been proposed as treatment options.

In our illustrative example, a personalized trial scenario for treating CLBP administered yoga and massage in a within-subject cross-over design. The outcome variable was the number of daily steps (Hendrick et al., 2010), assessed objectively with a wearable Fitbit activity monitor. The primary hypothesis was that both yoga and massage treatments would significantly increase the CLBP patient’s walking steps over their baseline compared to usual care. We had no hypothesis about whether massage versus yoga had differential efficacy. In light of the HTE found with other therapies, we expected the comparative effectiveness and degree of response to yoga and massage would vary across patients.

In Section 2.1, we will briefly go over the design of the CLBP trial. In Section 2.2, we will expand on the imputation of missing data in the step count data. The analysis of the step count data will be summarized in Section 2.3. In Section 3, we discuss the outcome of the trial and compare the individual results with a pooled analysis. The R shiny app will be described in detail in Section 4. We conclude this article with a discussion in Section 5.

## 2. Methods

### 2.1. Design of the CLBP trial

A series of 60 randomized personalized trials were conducted. This means each of 60 patients suffering from CLBP participated in their within-subject, multiple time-period cross-over trial testing the effects of Swedish massage and yoga versus usual care on physical activity (i.e., walking steps). All patients received a series of treatments of yoga and massage through Zeel (a commercial wellness service). Zeel allows participants to book in-home one-on-one yoga sessions with a certified yoga instructor. Yoga poses were selected based on those previously used by (Sherman et al., 2005) in a study assessing the effect on CLBP. Patients could also book in-home massages with licensed massage therapists through Zeel.

**Figure 1.**
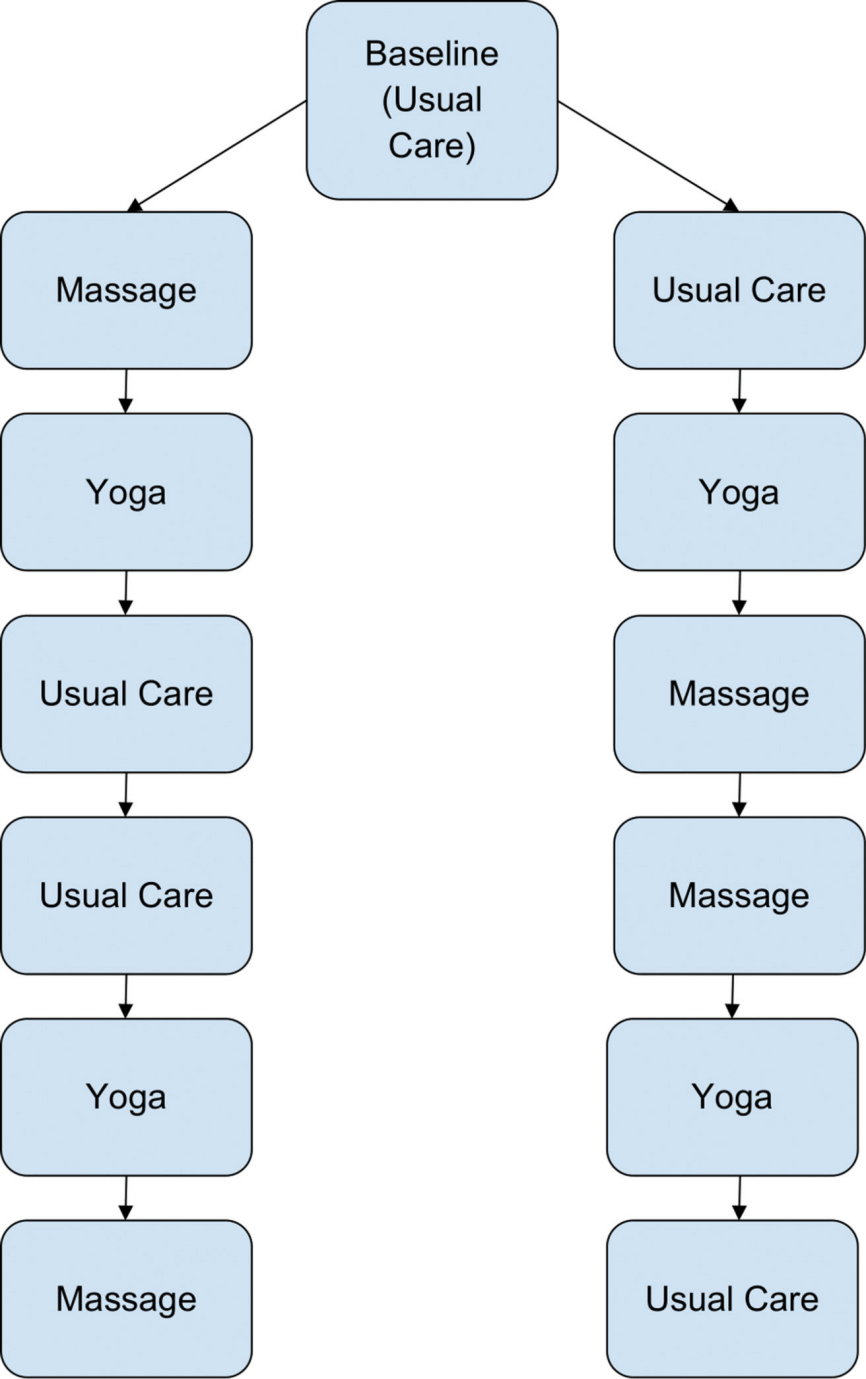
Flowchart of the treatment assignments.

Patients who met inclusion criteria underwent a baseline assessment period of 2 weeks (see Figure 1). During this period, patients were asked to wear a Fitbit Charge 3 activity-monitoring device that collected minute-by-minute step count data 24 hours a day. During the 2-week baseline period, patients were discouraged from receiving yoga and/or massage treatments. Adherence to wearing the Fitbit device was assessed during this 2-week baseline period. Those patients who did not achieve a minimum of 80% adherence (at least 11 of 14 days) during baseline were not continued to the intervention phases. The remaining patients were randomized to one of two different treatment sequences, which are depicted Figure 1. Two different sequences of the treatments were created to achieve balance in the assignment of treatments over time so that treatment effect estimates were unbiased by time-dependent confounders. Patients were randomized 1:1 to the treatment sequences (i.e., 30 participants in each treatment sequence).

As shown in Figure 1, the treatment sequences were designed to deliver two intervention arms (massage and yoga) and a usual care arm (no intervention) in a multiple-crossover design of six treatment blocks. Each treatment block lasted a total of 2 weeks. During intervention treatment blocks, patients were instructed to request Zeel to book two 1-hour sessions of in-home Swedish massage (massage treatment blocks) or two 1-hour sessions of in-home yoga (yoga treatment blocks) each week, at least 48 hours apart. No treatment was provided to patients during the usual care treatment blocks; instead, patients were asked to practice the techniques they normally used to manage their CLBP. Participants were discouraged from receiving additional massage or yoga sessions outside of the eight massage sessions and eight yoga sessions delivered throughout the trial.

#### 2.1.1. Recruitment and Study Population

Study participants were primarily recruited via emails sent to all employees at Northwell Health, the largest private health care employer in New York state. The email invited people with CLBP to participate in a personalized trial. Other recruitment strategies included referrals from Northwell Occupational Health Services (OHS), social media advertising, flyers distributed to Northwell Health facilities, and information presented at Northwell Health Wellness events. Those who were interested were asked to complete an online screening measure about both inclusion and exclusion criteria for the trial. If the individual was deemed to be eligible, an electronic consent form and additional information were provided.

The inclusion criteria for a participant included the following:

- Age ≥ 18
- Fluent in English
- Able to regularly access an email account and a smartphone
- Experiencing symptoms of lower back pain for ≥ 12 weeks
- A self-reported pain intensity > 8 on the PROMIS pain intensity scale (The PROMIS pain scales version 1.0 was used to measure the intensity of pain symptoms (Revicki et al., 2009) and interference (Amtmann et al., 2010) with daily life due to pain symptoms over the past 24 hours.)
- Able to receive therapeutics (2x per week; between 8 am and 10 pm)

Persons who met any of the following criteria were excluded:

- Pregnant women
- Weight ≥ 500 lbs
- History of spinal surgery
- Complex back pain
- History of a serious mental health condition or psychiatric disorder
- History of opioid use disorder or current opioid users
- History of treatment for any substance abuse
- Current physical activity restrictions or previously advised that yoga or massage is unsafe for their condition
- Planned travel outside of the United States within the treatment period
- Planned surgery/procedures within 6 months of recruitment

### 2.2. Imputing Step Count

Each participant was asked to wear a Fitbit Charge 3 device 24 hours a day. For each participant, the Fitbit data were recorded in a minute-by-minute file with step count, heart rate, date, and time (1,440 minutes per day for 14 weeks). Step count is recorded as 0 instead of ‘NA’ during a non-wear period or if the device battery is drained. However, the heart rate data is recorded as ‘NA’ if a participant is not wearing the device or if the device battery is dead. Even though participants had a high adherence rate, wearing the Fitbit at least 80% of the time, the imputation of missing step count is vital for the analysis in the next section. Thus, we present a model for the imputation of missing step count data.

Let $Y_{ti}$ be the actual step count at time t (or minute t) on day $i,X_{ti}$ be the step count recorded at time t on day i by a device (in our example, the Fitbit Charge 3), $W_{ti}$ be the indicator of wearing the device at time t on day i, and $H_{ti}$ be the indicator of heart rate being recorded at time t on day i. Heart rate is recorded to check whether the participant was adherent to wearing the Fitbit. We assume a participant is wearing the device when the heart rate is being recorded (i.e., $H_{ti}=W_{ti}$). When a participant is wearing the device at time t on day $i\;(H_{ti}=W_{ti}=1$), the actual step count at time t on day i is the same as the recorded step count at time t on day $i\;(Y_{ti}=X_{ti}$). If a participant is not wearing the Fitbit Charge 3 at time t on day i (i.e., $H_{ti}=W_{ti}=0$) and $X_{ti}>0$, we assume the participant is carrying the device but not wearing it at time t on day i. In this scenario, $Y_{ti}=X_{ti}$.

However, when a participant is not wearing the device at time t on day $i\;(H_{ti}=W_{ti}=0$) and $X_{ti}=0$, we assume $W_{ti}⊥Y_{ti}$ and

$$E\left(Y_{t}|W_{t}=0\right)=E\left(Y_{t}|W_{t}=1\right),$$

where $Y_{t}$ is the actual step count at time t (or minute t) and $W_{t}$ be the indicator of wearing the device at time t. We assume $Y_{ti}$ can be modeled using a parametric model,

$$Y_{ti}∼Poisson\left(\lambda_{ti}\right),$$

$$log\left(\lambda_{ti}\right)=\alpha_{t}+\beta_{t}r_{ti}+\gamma_{t}z_{ti}+\epsilon_{ti}$$

where $\lambda_{ti}$ is the Poisson rate at time t on day $i,\;\alpha_{t},\;\beta_{t}$, and $\gamma_{t}$ are regression coefficients at time $t,\;r_{ti}$ is the weekday indicator (1 - weekday/ 0 - weekend) at time t on day $i,\;z_{ti}$ is the temperature (in Fahrenheit) at time t on day i, and $\epsilon_{ti}$ is the error term at time t on day i. Similar to (Cheung et al., 2018), we used both temperature and weekday indicators in model (1) because these variables are important predictors for step count.

We fit model (1) for complete data (non-missing) to obtain the regression coefficients and use the regression coefficients to impute the missing data. Finally, we fit the penalized spline curve to improve the smoothness of the imputed step counts.

### 2.3. Analysis of the CLBP trial

The treatment arms are not independent in personalized trials, unlike in traditional RCTs. In personalized RCTs, the data is collected continuously throughout the trial, thus time-series data need to be analyzed using a correlation structure (Shaffer et al., 2018).

We analyzed the daily step count data using generalized least square (GLS) regression. The GLS estimator of linear regression is a generalization of the ordinary least square (OLS) estimator. When the OLS estimator violates one of the assumptions of the Gauss-Markov theorem, namely that of equal variances, the GLS estimator is used. Note that,

$$Y=\left[\begin{array}{c} Y_{1}=\sum_{t=0}^{1440}Y_{t1}\\ \vdots \\ Y_{n}=\sum_{t=0}^{1440}Y_{tn} \end{array}\right]$$

from Section 2.2, where $Y_{ti}$ is the actual step count at time t (or minute t) on day i. In a standard linear model,

Y=Xβ+ϵ,

where Y is the n×1 response vector (actual daily step count matrix), X is an n×p model matrix, β is a p×1 vector of estimated regression coefficients (treatment effect—yoga, massage, usual care), and ϵ is an n×1 vector of errors. The OLS estimator of β assuming that $\epsilon ∼N\left(0,\sigma^{2}I_{n}\right)$ (i.e., the errors are uncorrelated),

$${\hat{\beta }}_{OLS}={\left(X^{T}X\right)}^{-1}X^{T}Y,$$

with the covariance matrix

$$Var\left({\hat{\beta }}_{OLS}\right)=\sigma^{2}{\left(X^{T}X\right)}^{-1}.$$

However, in time-series data, the errors from the regression model are unlikely to be independent. Generalized least-squares regression extends OLS estimation of the standard linear model by providing for possibly unequal error variances and correlations between different errors. Let $V=Var\left(\epsilon |X\right)$, where V is an n×n symmetric positive definite matrix. In traditional RCT, the n represents the number of subjects; however, in personalized RCT, the n represents the number of time points. In a GLS regression, the covariance matrix has the following structure:

$$V=Var\left(\varepsilon \right)=\left[\begin{array}{cccc} \sigma_{1}^{2} & \rho_{1,2}\sigma_{1}\sigma_{2} & \cdots & \rho_{1,n}\sigma_{1}\sigma_{n}\\ \rho_{2,1}\sigma_{1}\sigma_{2} & \sigma_{2}^{2} & \cdots & \rho_{2,n}\sigma_{2}\sigma_{n}\\ \vdots & \vdots & \ddots & \vdots \\ \rho_{n,1}\sigma_{n}\sigma_{1} & \rho_{n,2}\sigma_{n}\sigma_{2} & \cdots & \sigma_{n}^{2} \end{array}\right].$$

There is an invertible matrix, such that $V=WW^{T}$. If we multiply the regression equation by $W^{-1}$, we get

$$W^{-1}Y=W^{-1}X\beta +W^{-1}\epsilon .$$

Replacing $\bar{Y}=W^{-1}Y,\;\bar{X}=W^{-1}X,\;\bar{\epsilon }=W^{-1}\epsilon$, we get $\bar{Y}=\bar{X}\beta +\bar{\epsilon }$. Thus,

$${\hat{\beta }}_{GLS}={\left({\bar{X}}^{T}\bar{X}\right)}^{-1}{\bar{X}}^{T}\bar{Y}={\left(X^{T}V^{-1}X\right)}^{-1}X^{T}V^{-1}\bar{Y}.$$

We can fit several time-series models with GLS or generalized estimating equations (GEE). The autoregressive model with order 1 [AR(1)] is one of the most frequently used models in personalized trials (Chen & Chen, 2014)(Kronish et al., 2019). (This allows for correlation between successive data points but in such a way that given the result of the most recent data point for a subject, those further back have no predictive value.) In an AR(1) model, the regression errors are assumed to be stationary. The errors are assumed to have the same expectation and the same variance:

$$\sigma =\sigma_{1}=\sigma_{2}=\cdots =\sigma_{n}.$$

In addition, the model postulates that correlations diminish as observations are farther apart in a specific form:

$$\rho_{a,a+k}=\rho_{a+k,a}=\rho^{k},\;-1\leq \rho \leq 1,\;a,k>0.$$

If both the values of ρ and $\sigma^{2}$ are known in an AR(1) model, $\hat{\beta }$ (treatment effect) can be easily obtained. However, these parameters are generally unknown.

## 3. Results

Although the original goal of the CLBP study was to randomize 60 participants to receive the protocol, all study activities were halted in March 2020 as a result of the COVID-19 crisis in New York. The analysis below describes the data of 26 study participants that were able to complete their intervention treatment blocks before the study was ended for infection-control purposes. Fourteen and 12 subjects were randomized to the left and right sides of the flowchart (Figure 1).

**Figure 2.**
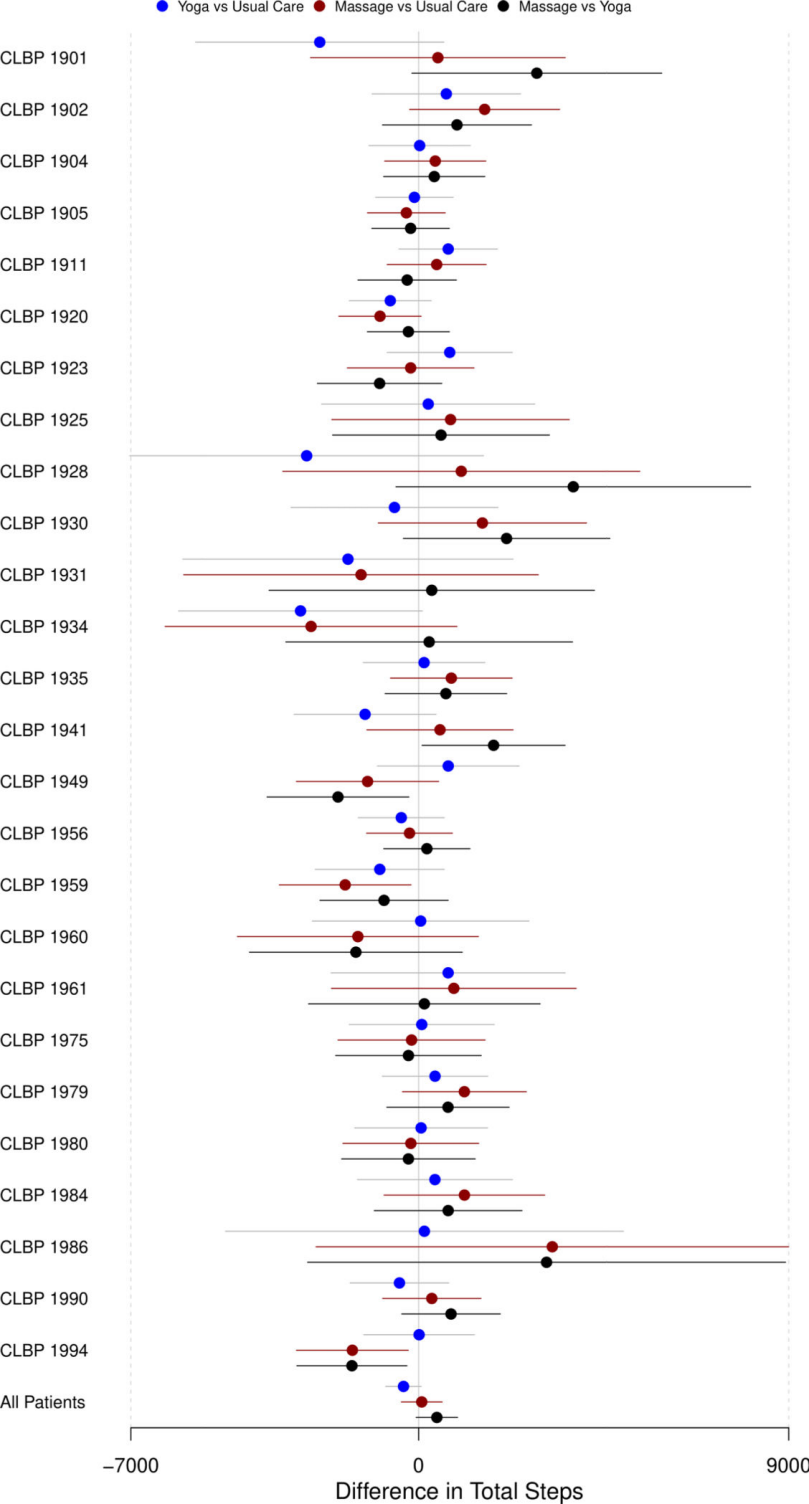
Forest plot comparing the difference in the total number of steps (per day) after imputation. The horizontal line being on the right or left side of the vertical line (in the middle) without intersection indicates the $\hat{\beta }>0$$\hat{\beta }>0$ and $\hat{\beta }<0$$\hat{\beta }<0$ respectively and the 95% CI of $\hat{\beta }$$\hat{\beta }$ does not include 0. On the other hand, the horizontal line intersecting the vertical line indicates that the 95% CI of $\hat{\beta }$$\hat{\beta }$ does include 0.

Figure 2 displays the summarized treatment effect obtained from daily step counts after imputation using GLS regression for all 26 patients. About 85% (22 of the 26) of the participants had no difference in daily step counts after imputation for both yoga and massage compared to usual care and yoga compared to massage (i.e., 95% [confidence interval] CI of $\hat{\beta }$ does include 0). Table 1 summarizes the outcomes of Figure 2. Table 1 shows the number of participants with no (95% CI of $\hat{\beta }$ does include 0), positive (95% CI of $\hat{\beta }$ does not include 0 and $\hat{\beta }>0$), and negative (95% CI of $\hat{\beta }$ does not include 0 and $\hat{\beta }<0$) effects that are statistically significant for daily step counts after imputation comparing yoga and massage to usual care and yoga and massage directly.

None of the participants had a higher daily step count ($\hat{\beta }>0$ & 95% CI of $\hat{\beta }$ does not include 0) after imputation when they received both yoga and massage compared to usual care and only two participants had a lower daily step count ($\hat{\beta }<0$ & 95% CI of $\hat{\beta }$ does not include 0) after imputation when he/she/they received yoga treatment compared to usual care. The final row in Figure 2 displays the pooled effect for the treatment effect for all patients. Figure 2 and Table 1 clearly show the HTEs for both yoga and massage compared to usual care in CLBP patients.

## 4. R shiny app

**Figure 3.**
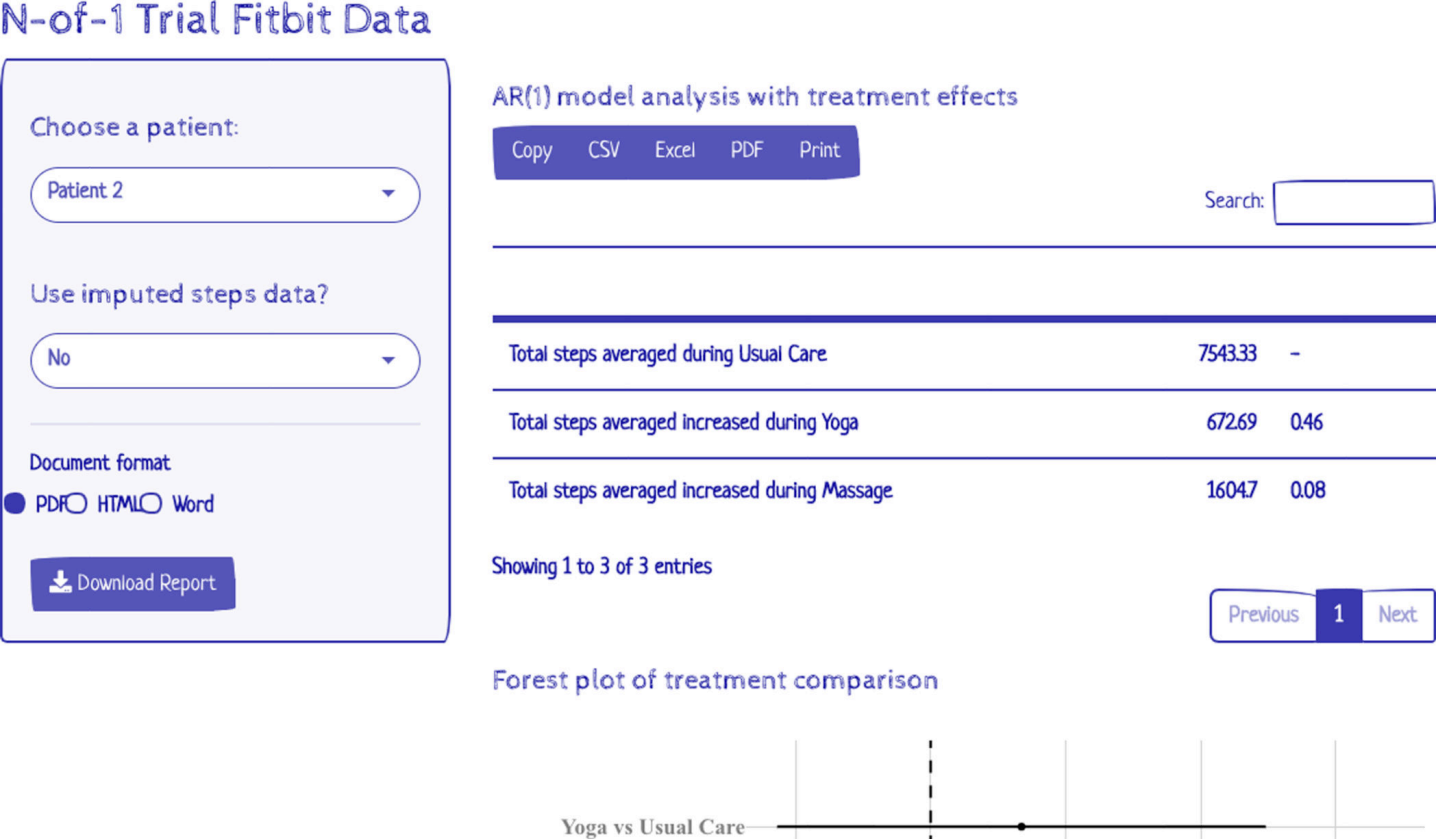
A screenshot of the R Shiny app that reports all the analyses for the step count data from the CLBP trial for each patient.

In Section 3, we focused on the summarized treatment effects obtained from the study and highlighted the HTEs for both yoga and massage compared to usual care. However, in personalized trials, the primary goal is to provide trial data for each patient to evaluate all treatment effects and select the best treatment option going forward. We developed a user-friendly R shiny app for reporting all the analyses for the step count data from the CLBP trial for each patient (video attached). The R shiny app is available at https://roadmap2health.io/hdsr/fitbit-shiny/ and the R code for the R Shiny app is available at https://github.com/ROADMAP-Columbia/fitbit-shiny (R Core Team, 2022). The Shiny app reports treatment effects from the GLS regression (table and forest plot), line graph, and boxplot of the step counts for each patient as shown in Figure 3. The analyses (gls regression, line graph, and boxplot) can be easily updated for both imputed and non-imputed daily step counts. The R shiny app can provide insight to patients about their health data.

The R shiny app can be used to generate analyses and descriptive statistics for the effectiveness of the outcomes of the two treatments utilized in this trial. By displaying results in this manner, research team members can easily identify the most effective treatments for each participant simply by clicking on the participant’s ID number within the R shiny app. Without the flexibility of this R shiny app, the research coordinator would need to consult with a statistician or attempt to run analyses themselves to generate these results. This would lead to more effort and the potential for errors.

In addition, the R shiny app runs based on the linked participant data set. This allows the analysis results to be continuously updated as participant data is added, easily generating both interim and final results for each participant through the course of the study. Further, the analyses utilized in this R shiny app can be easily modified to fit various N-of-1 designs to account for variations in the number of treatment blocks, duration of treatment, and other design elements essential to N-of-1 trials.

## 5. Discussion

In this article, we presented the design and analysis for a personalized trial treating CLBP patients who were administered yoga and massage in a within-subject cross-over design. The discussion focused on recruitment, including inclusion and exclusion criteria. The imputation of minute-by-minute Fitbit step data was explained and how personalized trials are typically analyzed using GLS because outcomes are dependent and susceptible to carryover effects. A summary of the individual trial data and forest plots (Figure 2 and Table 1) highlighted the HTE across individuals and indicates why N-of-1 trials can be more informative to patients and clinicians. Finally, we presented the R shiny app that can deliver statistical analyses to researchers. The app can provide a detailed report about trial results to each patient to learn about the effects of the treatments and to help them decide about the best option for them.

We acknowledge some limitations of our approach. We opted to use GLS with a temporal correlation of autoregressive order of 1 [AR(1)] over GEE. The treatment effects from both models, GLS with a temporal correlation of autoregressive order of 1 [AR(1)] and GEE with unstructured correlation, produced almost identical results. The Poisson distribution used to model the imputation of (minute-by-minute) step count may not always be the ideal model for all types of data. A negative binomial distribution captures overdispersion. That is, a negative binomial distribution allows the conditional variance of the outcome to be greater than its conditional mean, which offers greater flexibility for model fitting (Chandereng & Gitter, 2020). The Poisson distribution is also not ideal for capturing zero scores, which can be handled by a zero-inflated Poisson model (Cheung et al., 2018). In the future, the imputation of the step count can be improved with semiparametric models.

The R shiny app can be used to securely transfer trial results to the participants. The ability to collect data instantly using wearable devices has eased the implementation of personalized trials. In the near future, we are planning to streamline the data analysis process that provides the trial results instantly once the trial is completed. We also plan to build a simplified version of the shiny app that helps trial participants understand their data and the effect of each treatment for additional outcomes of interest (e.g., pain) that will be measured (one app for everything). The ultimate goal is to provide a tool for the patients to gain insight into their data and select the best treatment based on a data-driven approach. The importance of analytics and computing platforms to understand health data cannot be overemphasized.

## Figures and Tables

**Table 1.** Number of participants with no (95% CI of $\hat{\beta }$ does include 0), positive ($\hat{\beta }>0$ & 95% CI of $\hat{\beta }$ does not include 0), and negative ($\hat{\beta }<0$ & 95% CI of $\hat{\beta }$ does not include 0) significant effects for daily step count after imputation comparing both yoga and massage to usual care and yoga and massage directly.

	Positive effect (higher daily step count)	Negative effect (lower daily step count)	No effect
Yoga vs Usual Care	0	0	26
Massage vs Usual Care	0	2	24
Massage vs Yoga	1	2	23
